# Gastrointestinal Neuroendocrine Tumor, Colon Adenocarcinoma, and Hyperparathyroidism: An Unorthodox Synchronism

**DOI:** 10.7759/cureus.3731

**Published:** 2018-12-14

**Authors:** Hafiz M Aslam, Shumaila M Iqbal, Faizan Faizee, Anum Nida, Madiha A Qadir

**Affiliations:** 1 Internal Medicine, Seton Hall University-Hackensack Meridian School of Medicine, Trenton, USA; 2 Internal Medicine, University at Buffalo / Sisters of Charity Hospital, Buffalo, USA; 3 Internal Medicine, Dow University of Health Sciences (DUHS), Karachi, PAK; 4 Internal Medicine, Jinnah Sindh Medical University, Karachi, PAK

**Keywords:** colon adenocarcinoma, neuroendocrine tumor, hyperparathyroidism

## Abstract

Synchrony of colorectal adenocarcinoma with neuroendocrine tumor (NET) and hyperparathyroidism with colon adenocarcinoma is a rare entity, reported in a handful of cases. We authors would like to report a rare case of coexistence of poorly differentiated colon adenocarcinoma, NET of appendix and hyperparathyroidism. A 43-year-old Caucasian female was diagnosed with metastatic colon adenocarcinoma involving the caecum and appendiceal orifice. The resected specimen also confirmed presence of concurrent well-differentiated NET in distal half of appendix. For this patient, serum chemistry was significant for severe hypercalcemia with elevated parathyroid hormone and fairly normal range parathyroid hormone-related peptide. Importantly, association among the aforementioned conditions remains elusive and warrants further research; nevertheless, surveillance procedures could be performed in patients, if a correlation exists at all.

## Introduction

Colorectal carcinoma is the third most common malignancy in the United States. Adenocarcinoma is the most well-known subtype of the tumor, diagnosed by biopsies (>90%) with proximal colon being the most involved site [1-3]. Neuroendocrine tumor (NET) is another subtype, holding a meagre prevalence of 1–2% [4]. The concurrence of adenocarcinoma and NET might be of clinical significance. As of now, 18 reported cases have been accounted for it. Another association of colorectal adenocarcinoma exists with hyperparathyroidism, proposed in the current literature [5, 6]. The case discussed below documents an unorthodox synchronism: poorly differentiated adenocarcinoma of the colon, NET of appendix and hyperparathyroidism due to parathyroid gland hyperplasia.

## Case presentation

**Figure 1:**
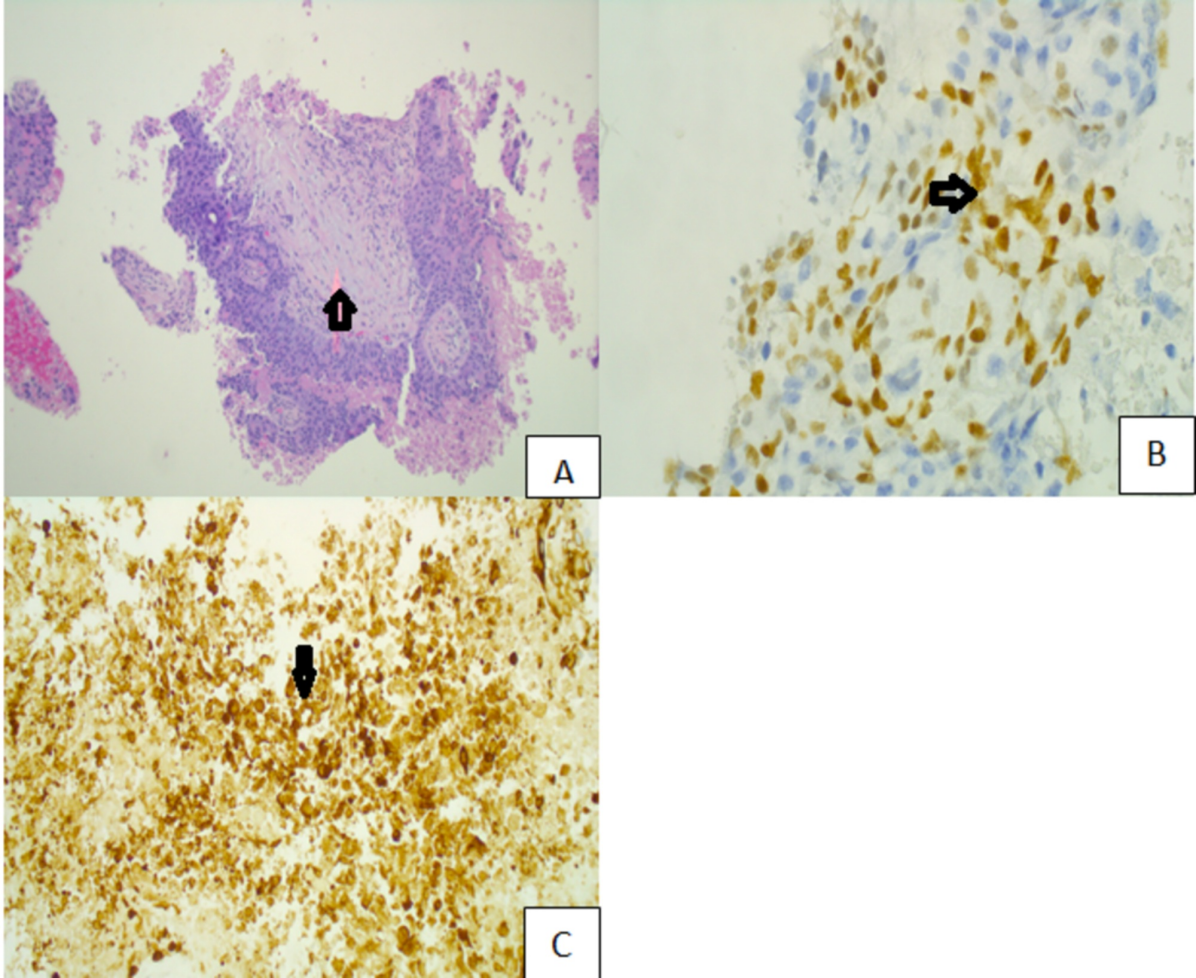
(A) Poorly differentiated adenocarcinoma from liver biopsy. (B) Positive staining for CDX2. (C) Positive staining for CK20.

A 43-year-old Caucasian female with a family history significant for colon cancer in paternal grandfather and uncle presented to the emergency department for a one-week history of generalized abdominal pain in association with nausea, unintentional weight loss of four pounds and shortness of breath. Initial blood work showed elevated liver enzymes and elevated total and direct bilirubin. Fecal occult blood test performed was positive. Computed tomography (CT) of abdomen/pelvis with contrast demonstrated ascites, retroperitoneal and periportal lymphadenopathy and hepatomegaly with extensive confluent masses in the liver parenchyma representing extensive metastatic disease. The scan was also noteworthy for dilated loops of small bowel, transverse colon distended and distal colon collapsed. Tumor marker, CA 19-9 was elevated. Core needle biopsy of liver exhibited poorly differentiated adenocarcinoma with immunohistochemical staining positive for CDX2 and CK20 (Figure 1), while being negative for PAX-8, CK7, p40, and TTF-1 indicating lower gastrointestinal tract being the primary site of origin for this metastasis respectively.

**Figure 2:**
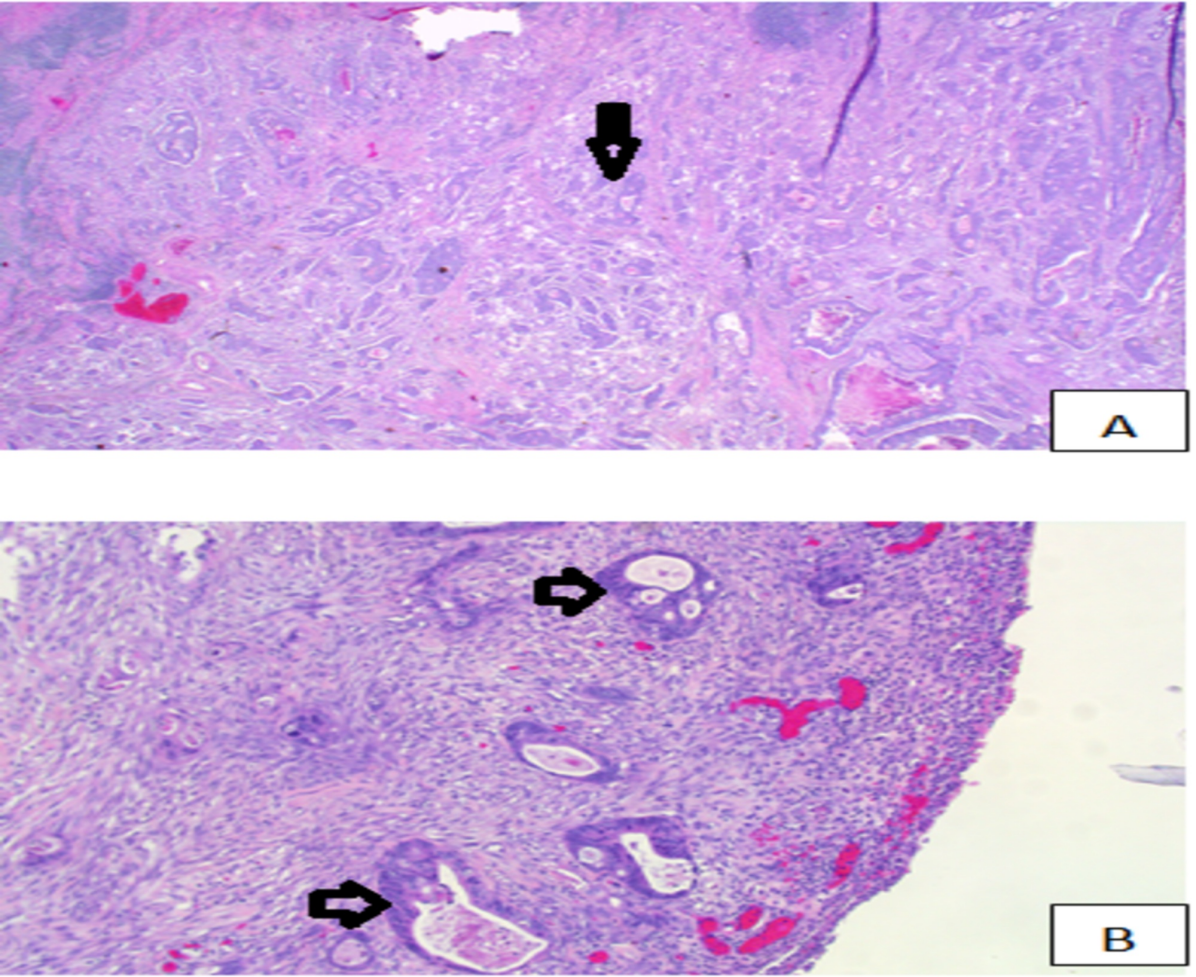
(A) Colon adenocarcinoma. (B) Colon adenocarcinoma invading parietal peritoneum.

Colonoscopy was performed which showed a large 5 x 6 cm fungating and friable obstructing mass in the cecum, 90 cm from the anal verge. Biopsies were taken and, results showed intramuscular adenocarcinoma in a background of tubulovillous adenocarcinoma (Figure 2). Right hemicolectomy with ileostomy was performed and resected specimen showed 4.7 cm high-grade (poorly differentiated) adenocarcinoma involving cecum and appendiceal orifice and perforating parietal peritoneum (Figure 2). Pathological staging of this tumor was pT4a, N1b, M1a (AJCC 7th edition) with low probability of microsatellite instability (MSI) found on immunohistochemistry.

**Figure 3:**
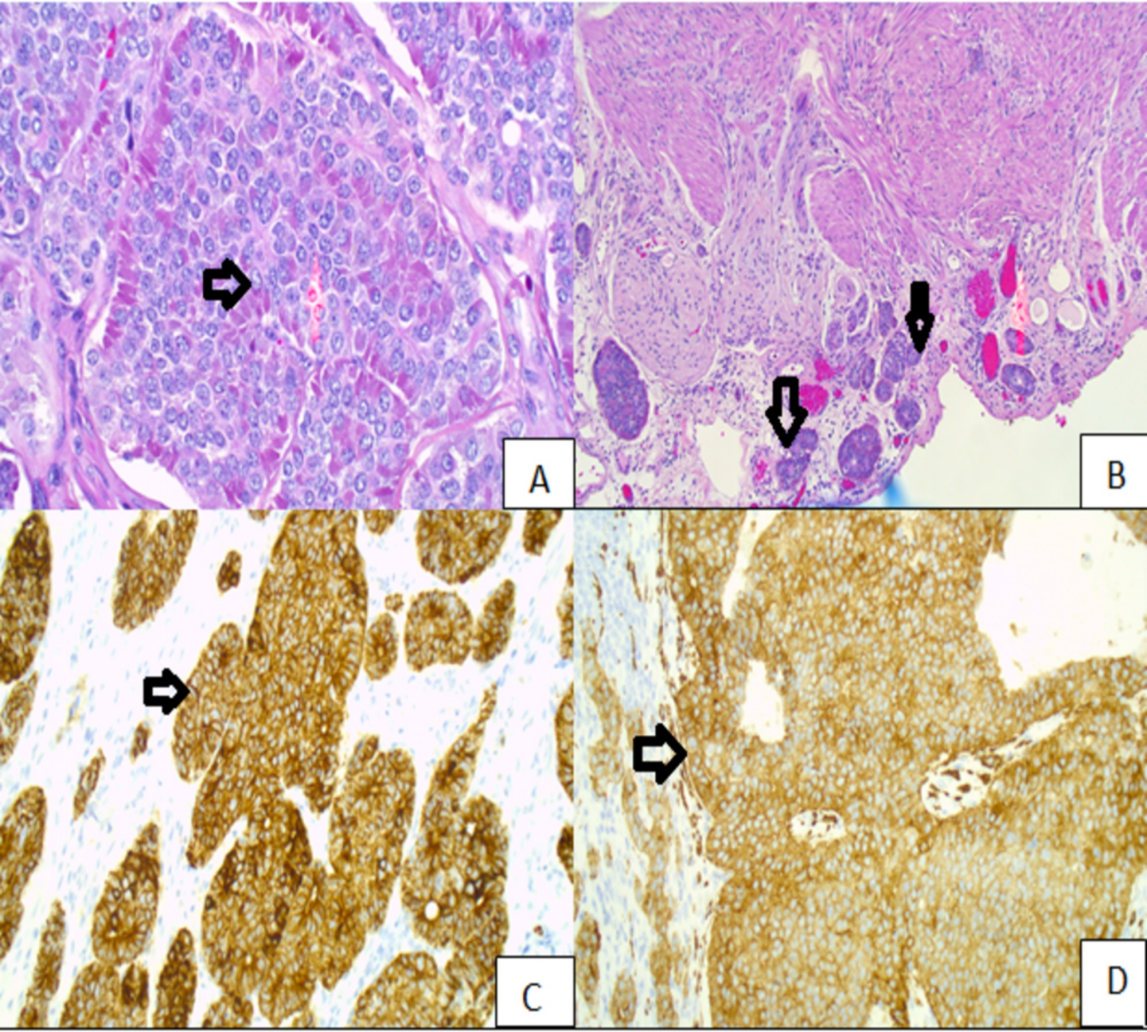
(A) NET. (B) NET invading visceral peritoneum. (C) Positive staining for pancytokeratin. (D) Positive staining for synaptophysin. NET: Neuroendocrine tumor

Surprisingly, surgical pathology report from resected specimen also showed a distinct 1 cm well-differentiated NET in distal half of the appendix invading visceral peritoneum (Figure 3) and staining positively for pancytokeratin and synaptophysin (Figure 3) with low Ki-67 proliferation Index (1%). Pathological staging of this tumor was pT4, N0 (AJCC 7th edition).

This patient’s blood chemistry was also significant for elevated calcium (16.5 mg/dl). Considering evidence-based association of hypercalcemia of malignancy by NET via production of parathyroid hormone-related peptide (PTHrp), further workup for hypercalcemia was performed. Surprisingly, PTHrP level was reported to be within fairly normal range (20 pg/ml) but parathyroid hormone (PTH) levels were found to be elevated (302 pg/ml). Ultrasound soft tissue neck showed parathyroid glands hyperplasia. 25-hydroxycholecalciferol level was at higher normal range 45 ng/ml. Patient's hypercalcemia was initially treated with intravenous fluid normal saline and calcitonin which resulted in decrement of serum calcium level to 11 mg/dl. Calcitonin was discontinued at that time. Couple of days later, calcium level went up again to 16 mg/dl. Calcitonin was added back into the medication regimen along with sensipar, pamidronate and lasix. The patient could not survive for further workup and later died from complications of the disease.

## Discussion

We unveil a circumstantial instance of simultaneous colon adenocarcinoma, hyperparathyroidism and intestinal NET in a 43-year-old female. Colorectal carcinoma is the third most commonly found cancer in men and the second most common cancer in women [4]. Adenocarcinomas make up 95% of every colorectal disease with the other 5% being mucinous and adenosquamous carcinomas [4]. Around 1% of colorectal cancer cases are NET that form within neuroendocrine cells which are involved in hormonal regulation. These tumors can develop in the pulmonary or the gastrointestinal tract.

There have been several cases reported that discuss the occurrence of the dual NET and colorectal disease with the principal case being accounted for in 1949 [4]. There are diverse proposed hypotheses to clarify the relationship between colon adenocarcinoma with gastrointestinal NET. For synchronous gastrointestinal NET and adenocarcinoma, one proposed clarification is the presence of common stem cells which additionally undergo comparative genetic transformations and give rise to various kinds of gastrointestinal malignancies [4]. As reported, a CK20 NET was identified in a patient who had simultaneous colorectal adenocarcinoma like our patient whose NET was likewise positive for CK20 [7]. A research study by Prommegger et al. demonstrated that the patients who had NET similarly had a secondary malignancy [8]. Intestinal NET is more often than not an indolent tumor if presents with concurrent colon adenocarcinoma, therefore research studies have recommended a requirement for stringent surveillance of non-adenocarcinomatous tumors of GI tract when an adenocarcinoma is identified respectively.

Another enigma was the presence of severe hypercalcemia in our patient. We authors were convinced that the severe hypercalcemia was a consequence of increased levels of PTHrP production by NET cells [9]. On the contrary, rigorous laboratory workup for hypercalcemia in this patient revealed normal serum levels of PTHrP along with escalated levels of PTH. The cause of elevated PTH was additionally bolstered with ultrasound neck findings significant for parathyroid gland hyperplasia.

For co-existing adenocarcinoma of colon and hyperparathyroidism, numerous hypotheses have been proposed in various research studies. One study suggests that colon adenocarcinoma manufactures a substance which has immunochemical attributes like parathyroid hormone owing to hyperparathyroid symptoms in the patients. The proposed mechanism of production of this substance is due to the derepression of coded genetic sequence that demonstrates synthesis of parathyroid hormone [6]. Other possible mechanism is the generation of parathyroid hormone by ectopic tissue or parathyroid glands, respectively [10]. Alternatively, high serum levels for 1,25- dihydroxycholecalciferol due to hyperparathyroidism enhance reabsorption of calcium by gut. Consequently, the calcium level in the lumen is decreased, leading to an elevated risk of colorectal cancer.

Till date, the possible relationship amongst colon adenocarcinoma, NET, and hyperparathyroidism remains elusive, and no conceivable clarification has been established which could open doors to explain the possible conjunction of these tumors at a molecular level. The peculiar associations, as presented in our case, warrant robust workup; thus investigations could be performed in patients if a correlation is speculated.

## Conclusions

The occurrence of concurrent colorectal adenocarcinoma with presence of NET and hyperparathyroidism is quite rare. We hope that our case and other similar reported cases in literature as discussed in this article prompt clinicians to conduct a surveillance colonoscopy to detect NET and investigate for parathyroid abnormalities when a patient is diagnosed with colorectal adenocarcinoma. This is to ensure early diagnosis and timely management.
